# Emerging human infectious diseases of aquatic origin: a comparative biogeographic approach using Bayesian spatial modelling

**DOI:** 10.1186/s12942-019-0188-6

**Published:** 2019-11-06

**Authors:** Soushieta Jagadesh, Marine Combe, Pierre Couppié, Paul Le Turnier, Loïc Epelboin, Mathieu Nacher, Rodolphe Elie Gozlan

**Affiliations:** 1 ISEM, UMR226, CNRS, Université de Montpellier, IRD, EPHE, 34090 Montpellier, France; 2 Equipe EPAT 3593 Ecosystèmes amazoniens et pathologie tropicale, Université de Guyane, Cayenne, French Guiana; 3 Service de Dermatologie, Centre hospitalier Andrée Rosemon, av des Flamboyants, 97304 Cayenne Cedex, French Guiana; 4 Unité des maladies infectieuses et tropicales, Centre hospitalier Andrée Rosemon, av des Flamboyants, 97304 Cayenne Cedex, French Guiana; 5 Centre d’investigation clinique (CIC Inserm 1424), Centre hospitalier Andrée Rosemon, Avenue des Flamboyants, 97304 Cayenne Cedex, French Guiana

**Keywords:** Outbreaks, Bacterial diseases, Buruli Ulcer, Leptospirosis, French Guiana, Urban environment

## Abstract

**Background:**

With the increase in unprecedented and unpredictable disease outbreaks due to human-driven environmental changes in recent years, we need new analytical tools to map and predict the spatial distribution of emerging infectious diseases and identify the biogeographic drivers underpinning their emergence. The aim of the study was to identify and compare the local and global biogeographic predictors such as landscape and climate that determine the spatial structure of leptospirosis and Buruli Ulcer (BU).

**Methods:**

We obtained 232 hospital-confirmed leptospirosis (2007–2017) cases and 236 BU cases (1969–2017) in French Guiana. We performed non-spatial and spatial Bayesian regression modeling with landscape and climate predictor variables to characterize the spatial structure and the environmental drivers influencing the distribution of the two diseases.

**Results:**

Our results show that the distribution of both diseases is spatially dependent on environmental predictors such as elevation, topological wetness index, proximity to cropland and increasing minimum temperature at the month of potential infection. However, the spatial structure of the two diseases caused by bacterial pathogens occupying similar aquatic niche was different. Leptospirosis was widely distributed across the territory while BU was restricted to the coastal riverbeds.

**Conclusions:**

Our study shows that a biogeographic approach is an effective tool to identify, compare and predict the geographic distribution of emerging diseases at an ecological scale which are spatially dependent to environmental factors such as topography, land cover and climate.

## Introduction

In recent years, rapid global and local environmental changes (e.g. deforestation, pollution, climate change) in the tropics have led to significant modifications in biodiversity [1]. Such rapid changes have underpinned alteration of host–pathogen patterns leading to more frequent and random emerging infectious disease (EID) outbreaks [2–4]. The direct and indirect consequences of these human-driven environmental changes result in the alteration of the geographic distribution of aquatic hosts and/or reservoir with a direct effect on the distribution of their pathogens (i.e. bacteria, viruses, parasites, fungi) [2]. Thus, the past and current geographical distribution of suitable habitats constrain pathogens survival and growth [3], leading to the redistribution of EIDs’ risk in local human populations. For example, deforestation has recently been linked to an increased risk of Buruli Ulcer (BU), a bacterial skin disease due to *Mycobacterium ulcerans* with high tropism for skin and causing severe ulcerations in humans [4]. Globally, over 20 years, the need for arable lands in the tropics in response to a rapid growing human population has led to a 28% decrease of primary forest [5]. Studies have demonstrated that small physical changes influenced by deforestation and climate in freshwater systems lead to significant restriction in the distribution of biota, altering the dynamics of these ecosystems [6, 7]. These freshwater habitats also act as a carrier of certain pathogens capable of causing human disease. Thus, rapid alterations in freshwater habitats can influence the exposure of the pathogen to humans, resulting in emergence of disease in a region over time. The global significance of EIDs caused by pathogens found freshwater habitats has been less studied in comparison to terrestrial ones [7–9] at an ecological scale. Outside malaria and cholera, there is little evidence linking spatial–temporal patterns of EIDs in human population and biogeographical freshwater drivers.

To construct a better understanding of biogeographical drivers influencing the local distribution of EIDs, the dynamics of human cases has to be analyzed in space and time in light of significant factors such as topography, land cover and climate. All three factors have direct implications in the availability of freshwater, the distribution in time and space of this water and on the type of aquatic habitats (i.e. flowing vs stagnant). It remains unclear if the emergence, transmission and distribution of EIDs in a region can be determined based on the spatiotemporal relationship between the disease incidence and these environmental factors. We hypothesize that the spatial structures of two EIDs caused by pathogenic bacteria occupying similar freshwater niche may overlap if a spatial pattern exists.

**Fig. 1:**
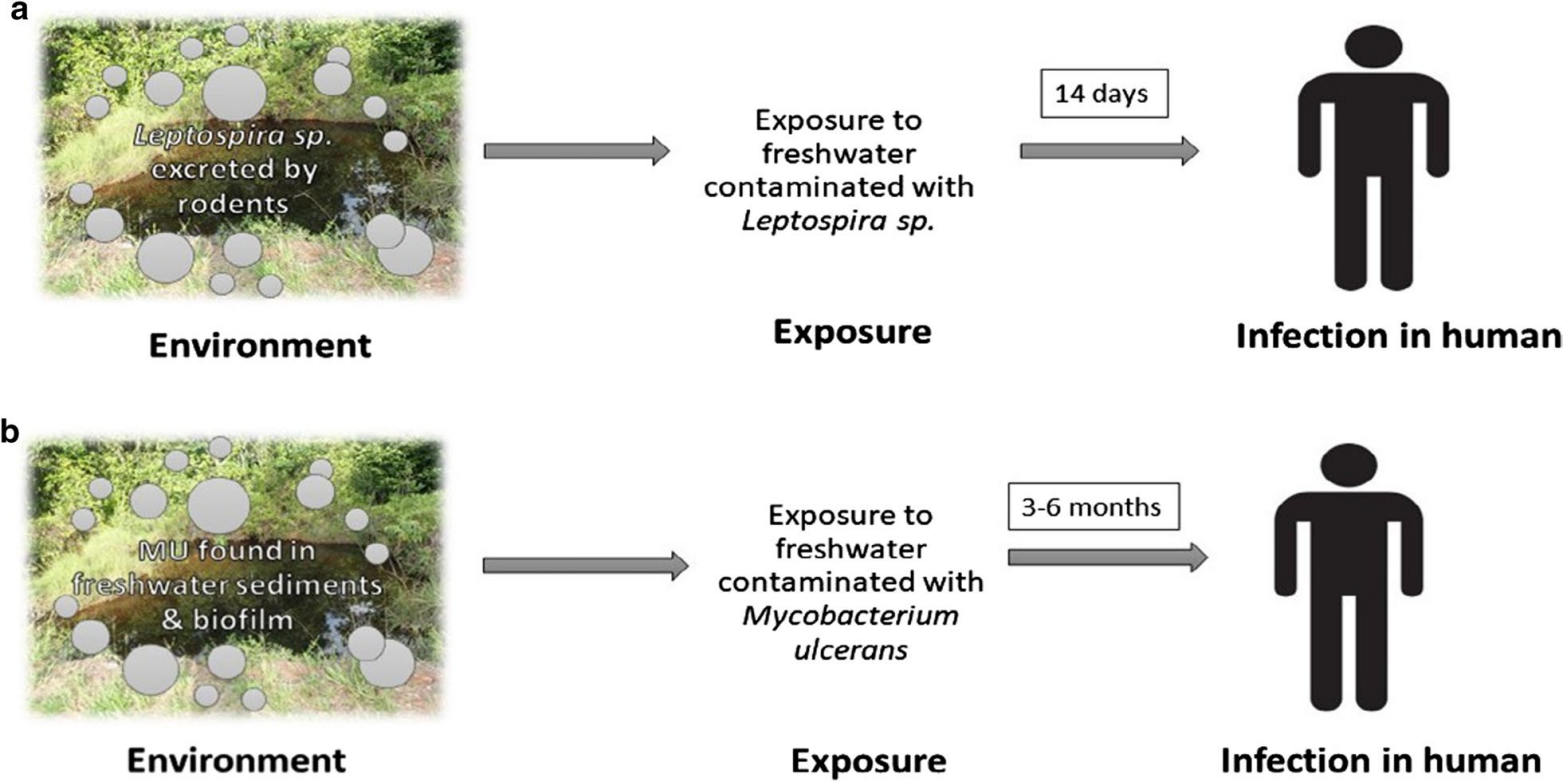
**a** Transmission dynamics of leptospirosis illustrating the host–pathogen–environment interface. **b** Transmission dynamics of Buruli Ulcer; MU represents *Mycobacterium ulcerans*

Here we used a couple of pathogenic aquatic bacteria, *Leptospira* spp., responsible for leptospirosis and *M. ulcerans* responsible of BU, as biological models to test our biogeographical predictions. These two infections are severe re-emerging diseases of epidemiological concern in humans in intertropical regions like French Guiana (FG), the area of study [10–12]. Although the transmission dynamics of the two bacteria are widely different, the mode of transmission to humans involves direct contact with aquatic habitats contaminated by the bacteria (Fig. 1). Leptospirosis is one of the most widespread zoonotic diseases as it occurs in temperate and tropical regions, and in urban and rural settings, dependent on the spatial distribution of its mammal reservoir, especially rodents such as *Rattus rattus* and *Rattus norvegicus*. Studies demonstrated that culturable pathogenic *Leptospira* were detected in soil for at least 16 days and in spring water for 28 days [13]. This suggests that the environment is not a multiplication reservoir but rather a temporary carrier for pathogenic *Leptospira*. While BU, on the other hand, is a generalist pathogen, globally more restricted in its spatial distribution to regions near wetlands and slow-moving rivers, notably areas prone to flooding in humid tropical and subtropical areas. *M*. *ulcerans* DNA has been detected in sediments, mud, detritus, biofilms, and aquatic invertebrates in still lentic and flowing lotic systems in the environment [14]. The transmission dynamics of BU still remains unclear, but is believed to be related to exposure to freshwater systems that contains *M. ulcerans* through abraded skin [14]. The public health response to the presence of pathogenic bacteria in the environment at present is reactionary. However, systematic surveillance of the pathogenic bacteria in the environment would aid in the prediction and control of outbreaks.

The overall aim of our study was to identify the patterns of leptospirosis and BU cases distribution and to quantify the local and regional biogeographic drivers underpinning such distribution. We hypothesized (1) that the local topography, land cover and climate spatially influenced the distribution of both diseases, and (2) that a distinct spatial pattern exists for the environmental drivers of both diseases, although spatial patterns may overlap as both infectious agents occupy similar freshwater niches. Such understanding is important to local Health Agencies in order to optimize local developments and habitat management as well as to incorporate EIDs risk in the decisions of local planers.

## Materials and methods

### Ethics statement

The study protocol was approved by Cayenne General Hospital authorities according to French ethical rules. The leptospirosis and BU database were declared to the Commission National Informatique et Libertés; numbers CNIL NO 2068308 and CNIL NO 3X#02254258 respectively following the requirements imposed by the French law. The database was anonymized and excluded from variables that facilitates identification of the patients. The leptospirosis and BU cases received appropriate treatment as per the French laws in public health.

### Study area

**Fig. 2:**
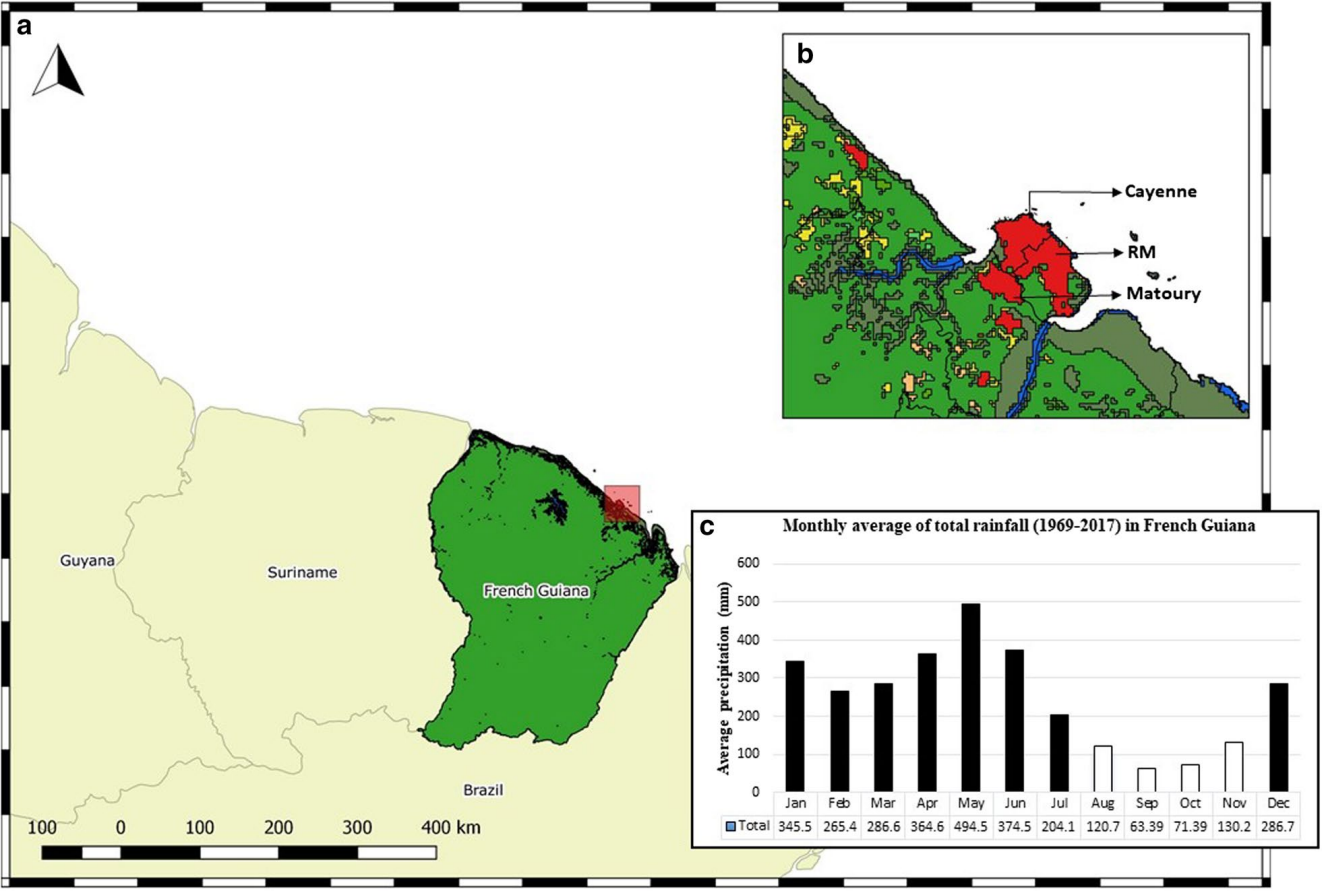
**a** Map of French Guiana showing the diverse land cover: primary forests in green, mangroves in olive, water in blue, urban area in red, shrubland in orange and cropland in yellow. Inset **b** Land cover map illustrating the proximity of primary forests to the urban regions of Cayenne, Remire-Montjoly and Matoury. **c** Insert is a graphical representation of the monthly average of total rainfall in millimeters (mm) from six meteorological station across FG; black bars represent the wet season and white bars dry season

Our study was conducted in French Guiana (FG), an overseas territory of France, located at 3.9339° N, 53.1258° W in South America (Fig. 2). The territory is divided in 22 administrative units termed as communes. Despite its land area of 83,534 km^2^, the territory has a low population density of 3.11/km^2^ with 72.78% (95 CI 0.726–0.728) of the total population living along the littoral region. Around 95% of the total land area is classified as primary rainforest forming a major portion of the highly biodiverse Guiana shield. The proportion of primary tree cover loss between the years 2001–2018 in FG was reported to be 0.6% and the loss per year (2011–2018) was 3200.69 trees/year. The land area also includes distinct areas of savannahs, wetlands, and coastal mangroves. The region is characterized by cyclic wet and dry seasons (Fig. 2).

### Diagnostic criteria and patient data

Cases of leptospirosis were defined as a case with ongoing symptoms that are compatible with the clinical description of the disease along with a positive PCR result or a fourfold rise in titer in 2 weeks measured by microscopic agglutination test (MAT) or a positive IgM ELISA. Cases from 2007 to 2014 (from January 1, 2007 to September 30, 2014) were validated by checking one by one all the medical charts. These cases were confirmed by having a positive PCR from blood or urine samples, cerebrospinal fluid and/or a MAT seroconversion with MAT titers ≥ 100 and/or a fourfold increase in MAT titers on two consecutive sera samples, and/or MAT titers ≥ 200 and/or a positive MAT titer with IgM seroconversion or IgM elevated titer. Cases from 2007 to 2014 were used in a previously published paper where the methodology is described in details [15]. Cases from 2014 to 2017 (from October 1, 2014 to December 31, 2017) were added to this study. No medical charts were checked and the microbiological diagnosis relied on positive PCR on any fluid, and/or positive MAT and/or positive IgM [16].

A confirmed case of BU was defined as a probable case with a clinically compatible cutaneous or bone lesion meeting the WHO clinical definition of BU [17] and the detection of *M. ulcerans* in smear or by histological examination using Ziehl–Neelsen microscopy or/and *IS*2404 PCR.

In both cases, we excluded duplicate entries based on the address of the patient; to exclude patients with recurrent infection or reinfection. The age, sex, date of diagnosis and the spatial coordinates (Cartesian coordinates) of the patient’s residence were extracted from the central hospital database managed by the specialist in-charge. The cases were projected at WGS84/UTM21 onto a shapefile (.shp) and the same coordinate reference system (CRS) was maintained for all spatial and statistical analysis done.

### Non-spatial vs spatial modelling

To test the objectives of our study, we developed non-spatial and spatial models using bayesian regression to identify if there was a spatial structure in the distribution of the cases or if the cases occurred in random. The model achieving a better fit was chosen. We also analyzed the influence of spatial drivers such as landscape and climate on distribution in the models. Finally, we compared the significant models of both the diseases under study to delineate the similarities in the biogeography of two bacterial diseases of similar freshwater origins.

### Topography

A one arc-second digital elevation model (DEM) of 30 m resolution, derived from NASA Version 3.0 Shuttle Radar Topography Mission (SRTM) imagery available in the United States Geological Survey (USGS) website, was constructed using QGIS (version: 2.8, Las Palmas). As the SRTM Global 1 arc-second product from 2016 is void filled, no further processing was done prior to hydrological modelling. The mean, minimum, maximum measures of the elevation and topological wetness index (TWI) were derived for each spatial point from a 30 m resolution DEM. TWI is an index measure that illustrates the capacity of a region to accumulate water in presence of rainfall [18]. TWI is a function of local upslope draining through a certain point per unit contour length (*a*) and slope (tan*β*). We calculated TWI, ln(*a*/tan*β*), from DEM models using TOPMODEL, a runoff method created by Beven and Kirby using to detect flood prone regions. The index detects potential ponding areas, regions of increased soil moisture, and rainfall runoff. The mean elevation was categorized into nine levels: 0–5 m, 5–10 m, 10–50 m, 50–100 m, 100–200 m, 200–300 m, 300–400 m, 400–500 m, and 500–600 m above sea level to explore the environmental attitude threshold for both infections.

### Land cover data

To assess the relationship between land cover and disease incidence, we used the MODIS-based Global Land Cover Climatology data developed by the USGS [19]. The data is derived from the Collection 5.1 MCD12Q1 land cover type data and is based across a time period of 10 years (2000–2010). It provides the land cover classification with the highest confidence as validated by Broxton et al. The composite raster of French Guiana was cut from the global map, matched in scale and resolution to the topological raster and its land cover type was broadly classified into eight classes (Table 1) using QGIS. Due the persistent cloud cover over the trans-equatorial zone in the imagery prior to 2000, disease cases exclusively from 2000 to 2017 were used for the land cover model generation. To analyze the land cover surrounding the disease cases, spatial buffers of radius 2 km, 5 km and 10 km were constructed. The radii of the spatial buffers were chosen to represent the land use and the environment in proximity to the sample population. The proportion of the land cover class contained in the three buffers for each spatial point was extracted and the resulting land cover variables was used for the regression models. One of the limitations in our study is the lack of land cover data at 30 m resolution for the years prior to 2000. As a result, the BU data prior to 2000 could not be used in the construction of land cover models and restricted the construction of the model combinations for BU. We chose to restrict the time period of land cover BU model, to focus on the spatial drivers of disease emergence to test our hypothesis. Using topological, land cover, and climate grids at 30 m resolution eliminates the spatial discrepancy between the models and ensures the quality of the spatial data used.

**Table 1:** Proportion of each land cover in French Guiana

Land cover class	Proportion of total land covered (%)
1. Primary forest	96.38
2. Mangroves	2.14
3. Water	0.77
4. Shrub land (flooded and non-flooded)	0.28
5. Crop land	0.13
6. Mosaic forest	0.13
7. Urban	0.12
8. Grassland	0.04

### Meteorological covariates

The meteorological data including the monthly maximum temperature (T_max_), minimum temperature (T_min_) and monthly total precipitation for the year 1968 to 2017 were obtained from six meteorological stations distributed across FG. The data was provided by Météo France. The monthly variables matched the temporal resolution for the disease cases used for Bayesian modelling. Climate covariates were spatially interpolated from points to climate grids using an Inverse Distance Weighted (IDW) approach in QGIS. IDW uses the nearest neighbor interpolation method, which takes on the value of the closest sample [20]. However, using local interpolation might not show micro influences where neighboring data is not local enough. In our study we used meteorological data from six weather stations located in the most habited regions and across the country to mitigate the underestimation of micro influences. The spatial resolution of the climate grid models matched the DEM models to preserve the resolution through the multilevel modelling. The value of the pixel that fell under the points representing the spatial points in the shape file was extracted, resulting in monthly meteorological covariates for each spatial point. In addition, in the case of BU, for each spatial point, the meteorological data 1 to 6 months prior to the reported date were also included to account for the unknown time of exposure, incubation period, appearance of symptoms and delay in health seeking behavior. To illustrate, the observed climatic covariates for a case reported in mm/yy (m) at a specific spatial point (xy) is noted and the climatic observations 6 months (m^−1^, m^−2^, m^−3^, m^−4^, m^−5^ and m^−6^) prior to “m” are also included. The incubation period of leptospirosis is distinctly shorter i.e. around 14 days, and so lag time was not included for leptospirosis.

### Regression modelling

To identify the significant relationships between the two diseases and the various topographical, landscape, meteorological and demographic variables across the country, regression modelling was used. We used mixed effects Markov Chain Monte Carlo approach for developing non-spatial and spatial models. To achieve optimal power in the regression models, we generated random spatial points across a spatial polygon file of FG (water bodies were excluded) stratified by the proportion of population in each commune. The number of background points was optimized by power calculation using G power (version 3.1) and an a priori number of 500 spatial points (approximate 1:2 = presence:absence ratio) were generated as controls. The same set of background points were used in both leptospirosis and BU modelling. To address the temporal nature of the controls, dates in dd/mm/yyyy format were randomly generated for the spatial point across the time periods; from 2007 to 2017 for the leptospirosis dataset and from 1968 to 2017 for the BU dataset.

### Non-spatial models

A non-spatial generalized liner model (GLM) was tested on the 232 and 236 cases of leptospirosis and BU respectively, and the strength of their association with elevation, landscape and climate covariates. The predictor covariates for each case was indexed by location, K = {k_i_,…,k}, where each k is a vector recording of the longitude and latitude at UTM 21 N projection. The response variable y(k) was the presence or absence of disease at generic location k. The covariates for each response variable at k were recorded. Simple logistic regression was done to check for the presence of association between the presence of case and each covariate. We then used a Markov Chain Monte Carlo (MCMC) sampler for Multivariate Generalised Linear Mixed Models to establish the relationship between the dependent variable and the covariates introduced as fixed effects. The “MCMCglmm” package was used for analysis [21]. The DIC was extracted from the models for comparison with the spatial models.

### Spatial models

All models were generated using the “binomial” family of spGLM function from spBayes R package. The model parameters were estimated using MCMC methods utilizing an adaptive Metropolis (AM) algorithm with a 43% acceptance rate [22]. The starting coefficient values and the beta tuning were obtained from the non-spatial logistic regression models. The predictor variables not significant in the logistic and MCMC regression non-spatial models were not analyzed in spatial modeling. Both the spatial and non-spatial models for each disease were tested individually for elevation and TWI under topographically variables, each of the land cover predictors, and climatic covariates such as maximum, minimum temperatures and total precipitation.

### Model comparison and verification

Models were compared using the deviance information criterion (DIC) for the Bayesian models. The models with lower DIC values indicate better model performance during model comparison similar to the AIC values [23]. Prior and posterior predictive checks were conducted to ensure the robustness of the models. All statistical tests were set at the conventional 5% significance level. All statistical models are based on assumptions and the spatial models in our study are no exception. In the Bayesian spatial models used, data was assumed to have a spatial structure. This assumption was mitigated by conducting a preliminary spatial cluster analysis to confirm the presence of a spatial structure of the leptospirosis and BU cases. The cluster analysis was done using satscan R package. The area-level random effects are not assumed constant but is under the assumption that the outcome between two neighboring spatial points is more similar than that between two distant spatial points in Bayesian models. Statistical R packages and datasets used for each model are detailed in Additional file 1: Table S1.

## Results

Our results show that leptospirosis was widely distributed across FG, occurring in 18 of the 22 communes (Additional file 1: Table S2). The incidence of leptospirosis in FG was found to be 0.96 [95% confidence interval 0.8–1.1] per 1000 people during the period 2007–2017. BU was found to be restricted to the cities and towns along the coast, occurring in nine communes. During 1969–2017, the incidence of BU was found to be 1.9 [95% CI 1.7–2.2] per 1000 people in FG. The incidences of both diseases for each commune has been provided in Additional file 1: Table S2.

### Spatial vs non-spatial models

Our results demonstrate that most spatial models (9 out of 12; 75%) produced lower DIC values in comparison to the non-spatial models (Table 2). This illustrates a spatial dependence of leptospirosis and BU cases distribution in FG towards environmental drivers such as elevation, topological wetness index (TWI), land cover and climate. On comparison of the regression coefficients between the spatial and non-spatial models, we observed that the non-spatial models overestimated the significance of the environmental variables likely attributed to the violation of the basic model assumptions (Table 3).

**Table 2:** DIC value of the leptospirosis and BU non-spatial vs spatial models

Model	Disease dataset	Non-spatial model	Spatial model
Mean elevation	Leptospirosis	683.01	413.67
	Buruli Ulcer	626.81	542.97
Mean TWI	Leptospirosis	842.56	523.21
	Buruli Ulcer	875.98	676.46
Land cover at 2 km	Leptospirosis	665.18	574.21
	Buruli Ulcer	6.61	304.14
Land cover at 5 km	Leptospirosis	591.65	534.07
	Buruli Ulcer	16.73	301.28
Land cover at 10 km	Leptospirosis	626.14	512.82
	Buruli Ulcer	6.05	279.83
Minimum temperature	Leptospirosis	748.95	518.39
	Buruli Ulcer	928.31	674.07

**Table 3:** Comparison of predictor variables of the different statistical models: logistic, Bayesian non-spatial, and spatial models

Model	Disease dataset	Logistic regression	Non-spatial MCMC model	Spatial MCMC model
Mean elevation	Leptospirosis	(−)	(−)	(−)
	Buruli Ulcer	(−)	(−)	(−)
Mean TWI	Leptospirosis	(+)	(+)	(+)
	Buruli Ulcer	(+)	(+)	(+)
Land cover at 2 km	Leptospirosis	(−) primary forest	(−) primary forest	(−) primary forest
	Buruli Ulcer	(+) urban	(+) urban	(+) urban
		(+) cropland	(+) cropland	(+) cropland
Land cover at 5 km	Leptospirosis	(−) primary forest	(−) primary forest	(−) primary forest
		(−) mangroves	(−) mangroves	(−) mangroves
		(−) urban	(−) urban	X
	Buruli Ulcer	(+) urban	(+) urban	X
		(+) cropland	(+) cropland	X
Land cover at 10 km	Leptospirosis	(−) mangroves	(−) mangroves	X
		(−) primary forest	(−) primary forest	(-) primary forest
		(+) cropland	(+) cropland	(+) cropland
	Buruli Ulcer	(+) cropland	(+) cropland	(+) cropland
		(−) urban	(−) urban	X
		(−) mangroves	(−) mangroves	(−) mangroves
		(−) primary forest	(−) primary forest	(−) primary forest
Maximum temperature	Leptospirosis	X	X	–
	Buruli Ulcer	X	X	–
Minimum temperature	Leptospirosis	(+) 0 month	(+) 0 month	(+) 0 month
	Buruli Ulcer	(+) − 4 months	(+) − 4 months	X
Total precipitation	Leptospirosis	X	X	–
	Buruli Ulcer	X	X	–

The (+) and (−) indicate the positive or negative correlation of the significant coefficients and “X” denotes non-significant coefficients

### Topological models

The spatial elevation models of leptospirosis and BU had lower DIC values than the non-spatial elevation models (Table 3). Elevation over 10 m (10–50 m, 50–100 m and 100–200 m) were negatively correlated to the presence of leptospirosis in the geographical area [95% CI − 0.1759 to − 0.0505, − 0.1690 to − 0.0831, − 0.1178 to − 0.0443 respectively]. Similarly, elevation at 10–50 m was inversely associated with the presence of BU [95% CI − 0.0946 to − 0.0301]. TWI was found to be a spatially dependent environmental driver with a significant positive correlation with the disease positivity ([95% CI 0.1353 to 0.2461 and 0.0651 to 0.1245] for leptospirosis and BU respectively).

### Land cover

The spatial models of leptospirosis achieved a better fit than those of BU in comparison to the non-spatial models for predictor variables, land cover at 2, 5 and 10 km. At a spatial buffer of 2 km, the presence of leptospirosis was inversely related to the presence of primary rainforest [95% CI − 5.4550 to − 2.926]. While at buffer of 5 km, the presence of mangroves [95% CI − 7.5813 to − 2.277] along with primary rainforest [95% CI − 8.5772 to − 4.834] negatively influenced the distribution of leptospirosis. At a buffer of 10 km, primary forest [95% CI − 9.1840 to − 5.3070] remained a negative predictor of leptospirosis while presence of cropland [95% CI 5.5302 to 18.2710] was found to have a positive influence. The distribution of BU on the other hand was found to be spatially independent based on the DIC values. However, the confidence intervals of the non-spatial models were wide and so the spatial models are reported instead given their methodological robustness. At spatial buffers of 2 km, urban land cover [95% CI 1.6300 to 2.934] along with cropland [95% CI 4.1979 to 6.888] were found to be a positive predictor of BU incidence. No significant variables were observed at buffer of 5 km. The presence of mangroves [95% CI − 7.7912 to − 2.3001] and primary rainforest [95% CI − 6.7331 to − 3.4672] were inversely associated with the presence of BU disease.

### Meteorological covariates

Increase in the minimum monthly temperature (T_min_) was found to have a positive influence in the distribution of leptospirosis and BU. The leptospirosis spatial model demonstrated a significant association with the predictor variable, T_min_ [95% CI 1.128 to 1.339] at month zero while in the BU spatial model, the regression coefficient was not significant. The other meteorological variables, maximum temperature and total precipitation, were found not significant in the non-spatial models.

### Model combinations

For leptospirosis, the best fitting model combination was mean elevation, cropland at 10 km and T_min_ at month 0, i.e. the month of potential infection. BU cases from the year 2000 was used for the model combinations due to the lack of landscape covariates for the earlier years. The best model fit was found to be mean elevation, cropland at 10 km, and primary forest at 10 km.

## Discussion

To our knowledge, this is the first study to identify and compare the effects of biogeographic factors on the spatiotemporal distribution of two emerging bacterial diseases of aquatic origin at an ecological scale. Our main finding is that the statistical models demonstrate the significance of spatial structure in the distribution of the two diseases. The environmental covariates were found to significantly influence the spatial distribution of both diseases. The robustness of the spatial Bayesian models along with the narrow confidence intervals of the predictor variables and lower DIC values support our hypothesis that biogeographic factors influence the spatial distribution of the two diseases. The spatial structure of the two diseases correspond with the geographical distribution of *Leptospira* spp. and *M. ulcerans* in the environment, as described previously in FG [24].

The top ranked model combination, ranked based on DIC, for both diseases included low elevation, high TWI and cropland at 10 km. This can be partly attributed to a higher proportion of population living in river basins of low elevation which attracts human settlements providing easy irrigation for agriculture. However, the inverse association with increased elevation and the positive relationship with the TWI, demonstrates that both diseases show spatial predilection towards low-lying regions that are also prone to flooding. A prospective cohort study in Brazil showed that households at low elevation had a high leptospirosis infection risk [25]. WHO reports the increased propensity of leptospirosis outbreaks following floods as flooding facilitates the spread of the pathogen through proliferation of rodents which shed large amounts of pathogenic leptospires in their urine and thus increase the exposure to a susceptible population [26]. The positive relationship between cropland and leptospirosis incidence is congruent with previous studies on leptospirosis that demonstrated that croplands and associated farming practices also enhanced the rodent population leading to increased exposure with surface water and soil contaminated by rodent excreta [27–29]. Other studies showed that freshwater bodies near rainforests were hotspots for leptospirosis [30, 31], that is also congruent with our observation of leptospirosis in villages found on the banks of the river Maroni that is surrounded with primary rainforest. The river basin and croplands were found to serve as a network that spatially attracts and concentrate small mammals that are potential reservoirs to leptospirosis, thus maintaining the bacteria in the region.

In our study, the site of exposure to the leptospira was unknown and so we assumed that the patients were infected in the vicinity of their residence as demonstrated by various studies [32, 33]. This is however in contradiction to le Turnier et al. [15], who hypothesized that occupation such as gold mining in proximity to the primary forest was the likely source of infection in French Guiana. If that were the case, our spatial models would not demonstrate a significant spatial structure but rather a random occurrence of cases. Our results are further supported by a recent study from French Guiana [24], which carried out environmental microbiological sampling for *Leptospira* spp. in urban and rural areas in proximity to forests. The study observed that the bacteria were detected in modified urban ecosystems rather than in areas near forests.

**Fig. 3:**
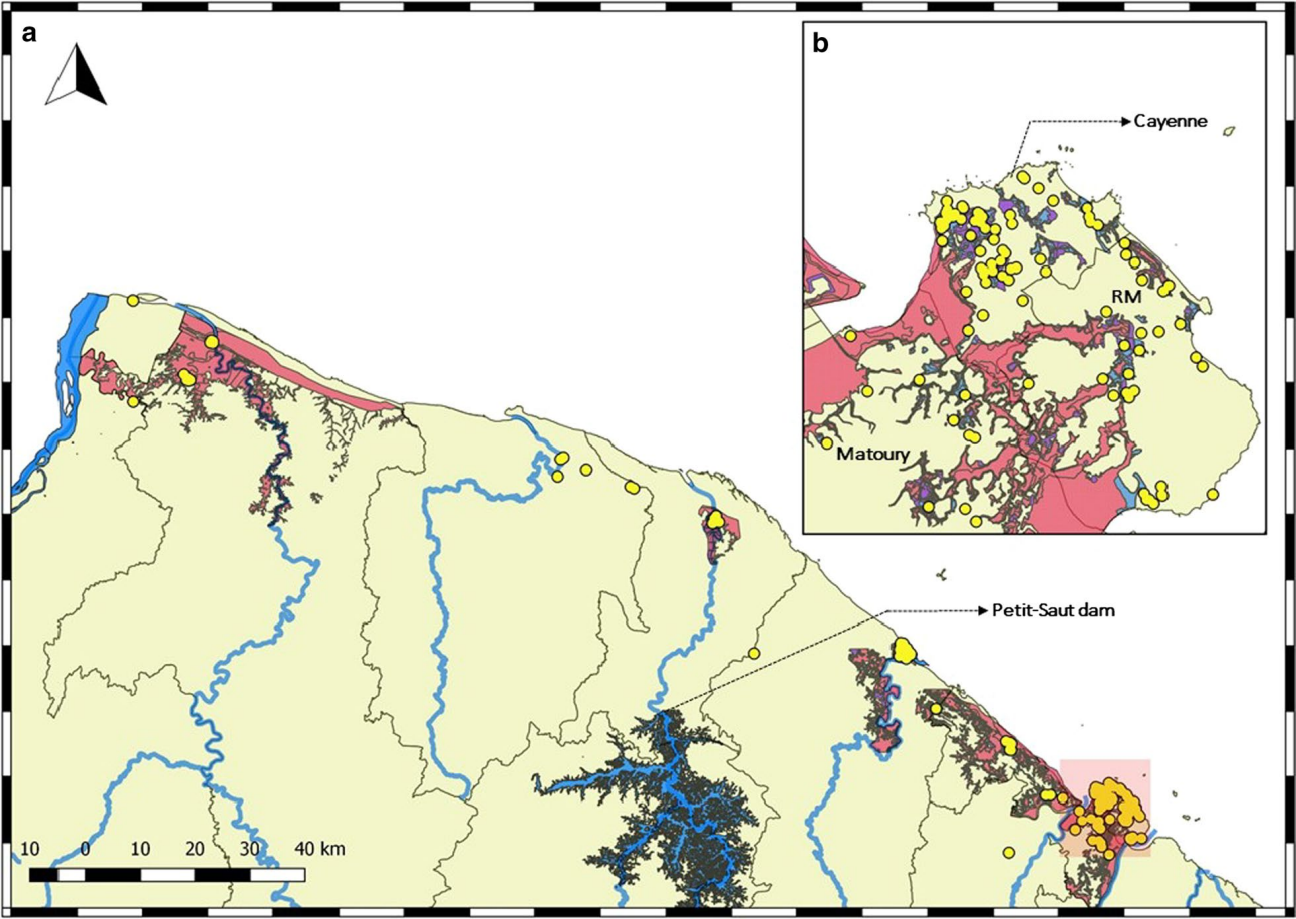
**a** BU cases (in yellow) occupying flood-prone regions along the banks of the major rivers in French Guiana. **b** BU cases in the flood-prone urban regions of Cayenne, Remire-Montjoly (RM) and Matoury with (1) zone red: regions in high risk of flooding, (2) zone blue: regions in average risk, and (3) zone purple: regions of low risk

Low elevation, river basins and agricultural activities were found to be also significant risk factors in the spatial distribution of BU. The BU cases were found occupy regions known to be prone to flooding in accordance to the cartographic regulatory document assessing the flood-prone areas in the commune known as the “plan de prevention du risque inondation” (PPRI) (Fig. 3). Studies have demonstrated the occurrence of BU disease outbreaks in West Africa and Australia associated with unprecedented flooding of rivers and lakes, damming of rivers and modification of wetlands into agricultural lands or recreational facilities [34, 35]. Flooding has been proposed to facilitate the transfer of *M. ulcerans* among aquatic reservoirs by providing a potential route for inter-water body dispersion [14, 34]. Previous studies in FG detected positive samples of *M. ulcerans* from freshwater bodies in the floodplains [36]. We observed a sharp decline in the incidence of cases in the region of Sinnamary following 1994, which corresponds to the construction of the Petit-Saut dam (Fig. 3) on the Sinnamary River. The building of the reservoir has been shown to influence flooding, in this case with a potential reduction of exposure to the pathogen in a susceptible population resulting in 11.38% annual decrease in incidence (p-value: 0.001) as supported by previous work from FG [11]. In West Africa, studies have demonstrated a positive association between BU incidence and agriculture [37–40], and a case–control study in Benin showed that farmers were associated with an increased risk of BU [37]. Overall, agricultural regions were found to have higher prevalence of BU due to an increase in nutrients favorable to biofilm growth and also decrease in dissolved oxygen content in surrounding freshwater bodies, which provides an ideal environment for *M. ulcerans*’s persistence [41]. Both bacteria occupying similar aquatic niche, despite of differing modes of transmission (Fig. 1), show similar relationships with topological risk factors, suggesting that low elevation, flood-prone regions near croplands are common risk factors for these specific bacterial diseases.

Interestingly our results demonstrate a positive relationship between urban land cover and BU incidence, which is in contradiction with other land cover studies conducted in West Africa on BU prevalence [37, 42]. The results from our datasets demonstrate that in the years following 2000, 79% of the new BU cases occurred in urban settings in comparison to 52% in the earlier years (79% vs 52%; p-value < 0.0001, 95% CI 14.96–37.94). This is supported by a recent study conducted in FG that also showed that modified urban ecosystems might favor BU emergence [24]. Also, the increasing number of cases in the urban regions of Victoria, Australia, provides a new perspective to BU incidence, which was previously thought to be restricted to rural areas [43].

The increased urban incidence of BU was demonstrated by the sharp rise of the disease in the sub-urban populations, namely Rémire-Montjoly (RM) and Matoury (Fig. 3) between the years 2002 and 2004. These regions were subjected to deforestation, modification of marshy lowlands into habitable areas during the early 2000s, as evidenced by the increase in annual rate of population growth by 3.3 and 7.8 between the years 1990–1999, 1.9 and 4.6 between 1999–2010 in RM and Matoury respectively. It is worth noting that these regions are more habited due to increased urbanization along the coastal regions resulting in large proportion of susceptible population. However, in contrast, the regions near the coast in Benin were found to have lower than expected BU prevalence, which was attributed to an access of pumped water sources in urban settings [37]. We propose that flood-prone regions associated with an increasing naïve population is at risk to develop BU due to the persistent maintenance of the bacteria in the environment as seen in Australia [44, 45].

Whilst previous studies from FG, including a time-series analysis, report the influence of rainfall on both disease incidences [15, 46], our study being a spatial biogeographical analysis did not found a significant correlation between disease incidences and rainfall. We observed that increase in minimum temperature during the time of potential infection can influence the prevalence of the diseases. Our results establish a positive relationship between increase in T_min_ at − 4 months (m^−4^) prior BU diagnosis, which corresponds approximately to the time of potential infection (i.e. the incubation period of the disease) [47]. A study from Australia also reports higher BU disease incidence with T_min_ conditions, with BU occurrence associated with T_min_ at − 18 months. However, such a lag phase does not correspond to the incubation period of the bacteria [45]. By comparison, leptospirosis was found to have a similar association to T_min_ during the month of diagnosis. Increase in T_min_ (1 °C) at a lag of 11 weeks was significantly associated with the increase in leptospirosis cases in the Republic of Korea [48]. In our study, the association between increase in T_min_ during the potential exposure period and disease incidence demonstrates that increase in minimum temperature plays a significant role during infection. It is interesting to note that studies on climate change report warming trends in minimum temperatures (T_min_) over time due to greater heat accumulation with consistent T_min_ increasing more than T_max_ [49]. This establishes an indirect connection between climate change and increasing incidence of both diseases. However, a time-series analysis will be needed to analyze this relationship further and to forecast potential implications in the future emergence of both diseases under scenarios of climate change in FG. This research has demonstrated that two aquatic diseases of bacterial origin are spatially dependent at an ecological scale and a biogeographic approach is important in identifying the factors influencing the disease emergence and maintenance in a region. This approach is especially useful when information on the host and pathogen distribution are unavailable.

## Conclusions

On comparing the environmental factors influencing the spatial distribution of two aquatic bacterial diseases of freshwater origin, we conclude that low-lying regions prone to flooding with near-by agricultural land and increased minimum temperature during the time of infection were found to be at risk for the increased incidence of both diseases. The trends of positive population growth rate in the urban regions of FG predict that deforestation and habitat fragmentation will continue to accommodate the needs of the growing population. Such human-driven regional environmental modifications along with global climate change affects vulnerable freshwater systems resulting in increased host–pathogen contact and ensure the maintenance of the aquatic bacteria at an ecological scale. Based on our results, we recommend the following to reduce the incidence of the two disease in developing tropical regions:

i. Better urban planning by construction in regions of low flood risk or low TWI, calculated from global satellite imagery. Regions of high flood risk need better drainage systems that would decrease human-pathogen contact.
ii. Croplands to be developed further away (over 10 km) from the population would also reduce human contact with rodents and with aquatic systems favorable for *M. ulcerans* and *Leptospira* sp.
iii. Increased minimum temperature during the time of infection signals the play of global environmental factors i.e. climate change. Climate change to be tackled at global scale to reduce the risk of disease emergence in tropical regions.
iv. Finally, conducting passive disease surveillance and measuring disease risk using biogeographic approach in regions where data on pathogen and reservoirs is scarce, is useful in the prevention and control of diseases.

## Supplementary information

**Additional file 1: Table S1.** Datasets and statistical models used in the study. **Table S2.** Incidence of Leptospirosis and BU in the communes of French Guiana. The significant numbers are given in bold.

## Data Availability

The clinical datasets were curated and maintained by authors PC, LE, PL, and SJ. The datasets analyzed are available upon request to the corresponding author.
