# Supplementary material for: Emerging human infectious diseases of aquatic origin: a comparative biogeographic approach using Bayesian spatial modelling
Source: Int J Health Geogr. 2019 Nov 6;18:23. doi: 10.1186/s12942-019-0188-6 (PMC6833193; doi:10.1186/s12942-019-0188-6)
Supplement: Supplementary file 1 — Additional file 1: Table S1. Datasets and statistical models used in the study. Table S2. Incidence of Leptospirosis and BU in the communes of French Guiana. The significant numbers are given in bold. [file 12942_2019_188_MOESM1_ESM.docx]

**ADDITIONAL INFORMATION**

| **Model** | **Objective** | **Variables used** | **Time Period** | **Model** | **R package used** |
| --- | --- | --- | --- | --- | --- |
| Preliminary Cluster analysis | Detection of significant spatial structure | Leptospirosis and BU case as spatial points | Leptospirosis: 2007 - 2017  BU: 1969 - 2017 | Poisson regression | satscan |
| Topographic model | To correlate elevation and flooding to incidence of Leptospirosis and BU in non-spatial and spatial models. | Mean, minimum, maximum measures of the elevation and TWI calculated from DEM of 30m.  Leptospirosis and BU case as spatial points along with the randomly generated spatial points. | Leptospirosis: 2007 - 2017  BU: 1969 - 2017 | Non-spatial vs Spatial Logistic regression | Stats  MCMCglmm  spBayes |
| Land cover model | Identification of land cover type that flavors the incidence of Leptospirosis and BU in non-spatial and spatial models. | Proportion of land cover in spatial buffers of 2km, 5km and 10km radii around Leptospirosis, BU cases and random spatiotemporal points. | Leptospirosis: 2007 - 2017  BU: 2000 - 2017 | Non-spatial vs Spatial Logistic regression | Stats  MCMCglmm  spBayes |
| Meteorological model | To correlate climatic covariates to the incidence of Leptospirosis and BU in non-spatial and spatial models. | Interpolated from points to climate grids of 30m resolution using an Inverse Distance Weighted (IDW) approach  Leptospirosis (months 0&-1) and BU case data (months 0 upto -6) as spatial points along with the randomly generated spatiotemporal points. | Leptospirosis: 2007 - 2017  BU: 1969 - 2017 | Non-spatial vs Spatial Logistic regression | Stats  MCMCglmm  spBayes |

**Table S1:** Datasets and statistical models used in the study

**Table S2:** Incidence of Leptospirosis and BU in the communes of French Guiana. The significant numbers are given in bold.

| **Commune** | **Disease** | **Mean population over time period** | **Incidence per 1000 population** | **95% Confidence Interval (CI)** |
| --- | --- | --- | --- | --- |
| Cayenne | Leptospirosis | 57614 | **1.8** | 1.4-2.0 |
|  | BU | 41847 | **1.9** | 1.5-2.3 |
| Iracoubo | Leptospirosis | 1927 | 1.0 | 0.3-3.8 |
|  | BU | 1257 | **11.9** | 7.2-19.6 |
| Kourou | Leptospirosis | 25705 | 0.1 | 0.0-0.3 |
|  | BU | 14921 | **1.7** | 1.2-2.6 |
| Macouria | Leptospirosis | 10680 | 0.6 | 0.3-1.2 |
|  | BU | 4452 | 1.8 | 0.9-3.5 |
| Mana | Leptospirosis | 9597 | 0.2 | 0.1-0.8 |
|  | BU | 4150 | **8.9** | 6.5-12.3 |
| Matoury | Leptospirosis | 19915 | **2.4** | 1.8-3.1 |
|  | BU | 18359 | 0.76 | 0.5-1.3 |
| Montsinery Tonnegrande | Leptospirosis | 2345 | 1.7 | 0.7-4.4 |
|  | BU | 2473 | 0.4 | 0.1-2.3 |
| Rémire-Montjoly | Leptospirosis | 21627 | 1.1 | 0.7-1.6 |
|  | BU | 14118 | **2.55** | 1.8-3.5 |
| Sinnamary | Leptospirosis | 3100 | 0.3 | 0.1-1.8 |
|  | BU | 2160 | **9.72** | 6.4-14.8 |
| Camopi | Leptospirosis | 1697 | 1.8 | 0.6-5.2 |
| Grand Santi | Leptospirosis | 6017 | 0.3 | 0.1-1.2 |
| Maripasoula | Leptospirosis | 10165 | **1.9** | 1.2-2.9 |
| Papaichton | Leptospirosis | 6047 | 0.3 | 0.1-1.2 |
| Regina | Leptospirosis | 910 | **4.4** | 1.7-11.2 |
| Roura | Leptospirosis | 3157 | 1.6 | 0.7-3.7 |
| Saint Georges | Leptospirosis | 4029 | 1.2 | 0.5-2.9 |
| Saint-Laurent-Du-Maroni | Leptospirosis | 40984 | 0.2 | 0.1-0.4 |
| Saul | Leptospirosis | 153 | **6.6** | 1.2-36.1 |
